# Carotid atherosclerotic disease

**DOI:** 10.1590/1677-5449.011703

**Published:** 2018

**Authors:** Cid José Sitrângulo, Erasmo Simão da Silva

**Affiliations:** 1 Universidade de São Paulo – USP, Faculdade de Medicina, Disciplina de Cirurgia Vascular e Endovascular, São Paulo, SP, Brasil.

 Treatment of atherosclerotic disease of the carotids has been the subject of constant debates and controversies over the years, as new knowledge and technological advances are incorporated alongside those previously established. Even though there are large numbers of good quality scientific articles published in the literature (multicenter randomized studies, cases series, literature reviews, and metanalyses, data from large-scale registers based on real clinical practice, and expert opinion on the subject), there is still margin for controversial opinions on some aspects of clinical or surgical treatments. This is because, from a clinical point of view, there are many considerations to be taken into account when making decisions on each specific case, because of the complexity and variability of the disease itself and of the therapeutic possibilities available. Therefore, atherosclerotic stenosis of the carotid bifurcation is a condition with respect to which the recommendations of evidence-based medicine sometimes come up against the sensitivity and experience of the treating physician. 

 Initially, it is worth noting that stenosis of the carotid bifurcation, the main focus of this article, is responsible for approximately 20% of cerebral ischemia cases. However, there are reports putting its prevalence in the range of 6 to 40%, which could be because of coexisting factors (associated diseases) that contribute to a considerable number of neurological events of indeterminate origin. In addition to carotid stenosis, the patient may have cardiac disease (atrial fibrillation, valve disease, inactive cardiac areas after acute myocardial infarction – AMI), aortic arch disease, common carotid disease, intracerebral atherosclerotic disease, etc. The prevalence of carotid stenosis increases with age and, at 65 years of age, 5% of people have carotid stenosis exceeding 50%, while the incidence among men is double that among women. With relation to the risk of people with carotid stenosis suffering a stroke, if stenosis is less than 50%, the likelihood is less than 1% per year, but if stenosis exceeds 50%, the likelihood is in the range of 1 to 5% per year. 1

 The relationship that exists between carotid stenosis, coronary atherosclerotic disease, and peripheral arterial occlusive disease of the lower limbs has considerable prognostic importance. Carotid stenosis is considered an important marker of cardiovascular risk and death from cardiac causes, to the extent that people with progressive carotid stenosis are at greater imminent risk of developing AMI than they are of future cerebral ischemic events. Rothwell conducted a prospective study with patients who had had a cerebral ischemic event and quantified the risk of developing AMI within 5 years at 10 to 25% and the risk of death from cardiovascular causes (except stroke) in 5 years as up to 15%. 2 Notwithstanding, although there is no consensus with relation to the severity of carotid stenosis, it is believed that the greater the degree of stenosis, the greater the possibility of a neurological event. There is also no consensus on whether the worse the degree of carotid stenosis the greater the possibility of manifestations of severe or moderate coronary disease. 

 Treatment options for atherosclerotic carotid stenosis start with clinical treatment (best medical therapy), which implies vigorously attacking the most important risk factors for atherosclerosis (smoking, arterial hypertension, diabetes, and dyslipidemia), combined with systematic physical activity, healthy diet, and prescriptions for platelet antiaggregants and statins at high doses. In cases with more accentuated carotid lesions, endarterectomy or angioplasty and stenting may be necessary. When opting for these interventional treatments, several factors should be taken into consideration, such as age, sex, principal comorbidities, especially cardiac, renal, and pulmonary diseases, and the anatomic and pathological conditions of the aortic arch. Particularly with relation to age and sex, there are certain controversies related to the choice of best treatment option. Among elderly patients, arterial tortuosity is generally greater and the atherosclerotic process tends to be more intense, provoking greater calcification of the aortic arch and of the carotids themselves, thereby increasing the risk of embolic accidents during angioplasty. Among females, in whom the natural history of atherosclerotic disease can be more aggressive and who habitually have smaller caliber arteries, there is an increased likelihood of postoperative restenosis in cases treated with endarterectomy, which constitutes one of the recommendations for using an arterial patch. 

 To a certain extent, the randomized, controlled, multicenter studies of symptomatic and asymptomatic carotid stenosis conducted in the 1980s and 1990s in the United States (NASCET – symptomatic and ACAS – asymptomatic) and in Europe (ESCT – symptomatic and ACST – asymptomatic), led to a standardization of management of severe stenosis of the internal carotid arteries. Overall, these studies agreed that both for cases of symptomatic and for cases of asymptomatic stenosis, endarterectomy was superior to clinical treatment for prevention of future ischemic cerebral events within 5 years. 

 With development of endovascular surgery in the carotid territory, a new question came to the foreground in clinical practice: would use of angioplasty and stenting (a procedure that is known to be less invasive than endarterectomy) produce similar, or at least not inferior, results to endarterectomy? Further multicenter studies were conducted in the 2000s and 2010s in the United States (CREST was the most important) and Europe (ICSS, among others) with the objective of answering this question and providing a foundation for consolidating the indications for each of these techniques. In contrast with the studies conducted in the 1980s and 90s, this time the results of the most important studies were to a certain extent contradictory: while the conclusions of the CREST study reported similar results for endarterectomy and angioplasty with stenting for prevention of future cerebral ischemic events, a large proportion of the European studies concluded that angioplasty with stenting was inferior to endarterectomy, especially in cases of symptomatic carotid stenosis. It is interesting to note that the American Heart Association (AHA), strongly inspired by the results of CREST, published guidelines for treatment of severe carotid stenosis recommending endarterectomy and angioplasty with stenting equally. The recent update to the AHA guidelines has maintained these initial recommendations, despite European authors disputing them. 

 More recently, a new multicenter study has been started, led by the University of Oxford, in the United Kingdom, and with the participation of several European universities, plus the vascular and endovascular surgery service at the Universidade de São Paulo Medical Faculty’s Hospital das Clínicas. The ACST-2 study, which has already enrolled more than 3,000 randomized cases, compares the results of endarterectomy and angioplasty with stenting in cases of asymptomatic carotid stenosis for prevention of future ischemic cerebral events. 

 In parallel with all of these discussions about the best interventional treatment method for severe carotid stenosis, in “real life” the number of indications of endarterectomy and for angioplasty with stenting in management of cases has been reducing over recent years. The most plausible explanation for this fact is recognition, in particular by clinicians, cardiologists, and neurologists, that, when patients are compliant, the clinical treatment (best medical therapy) currently proposed in its more aggressive and complete form can reduce the occurrence of stroke, especially in asymptomatic carotid stenosis cases. Many respected authors in the international literature have made observations to this effect. To add weight to these convictions, we can refer to work that has used imaging exams (Doppler mapping, magnetic resonance imaging, and angiotomography) to document the stability and even regression of atheromatous plaques with high doses of statins and rigorous control of atherosclerosis risk factors. 3 We must take into account the fact that in the early large-scale studies of carotid stenosis, use of statins was irregular and was not uniform. Nowadays, other complementary drugs can also be used in combination, when needed, to control cholesterol and triglycerides (ezetimibe, fibrates, niacin), and this may be a crucial factor in achieving more satisfactory results from clinical treatment. Aspirin is the most widely used of the platelet antiaggregants, although clopidogrel and ticlopidine are also options. Arterial blood pressure control can now involve use of hypotensive drugs with components that offer endothelial protection, angiotensin-converting enzyme inhibitors or angiotensin inhibitors. Early control of diabetes and quitting smoking are of fundamental importance, as are changes to lifestyle habits, with regular and permanent physical activity. With this combination of integrated therapeutic measures, progression of atherosclerotic disease can be contained, reducing its effects on carotid and cardiac territories and justifying the observation that nowadays clinical treatment is more effective than in the past. Additionally, the benefits of endarterectomy compared to clinical treatment that were observed in the initial studies of asymptomatic carotid disease were always borderline and based on higher rates of stroke with less incisive clinical treatments. Therefore, particularly for patients with high clinical or cardiological risk, clinical treatment for severe asymptomatic carotid stenosis is becoming more attractive. 

 However, optimal clinical treatment is frequently disrespected, since compliance is variable on the part of both clinicians and their patients. Additionally, the lifestyle changes are hard to achieve (stopping smoking, eating better, losing weight, exercising). The number of medications needed to treat risk factors is high and constant dose adjustment is needed, which requires many visits to the clinician. The ideal structure of consultations with professionals specialized in nutrition, psychology (smoking cessation) and nursing for pre and post-consultations has been defined and is adhered to in randomized, controlled studies, but not always in “real life”, especially not in poor countries. 

 Another interesting debate is related to reproducibility of the results of controlled studies in relation to results in “real life”. It is well known that the results of invasive procedures conducted beyond the ambit of randomized and controlled studies are inferior. It is also intuitive that, in contrast with controlled studies in which patients are allocated to treatments according to rigid and uniform selection criteria, many factors are present that would exclude patients from enrollment on studies and can cause more operative and postoperative complications, leading to inferior results to those observed in controlled studies. Furthermore, in controlled studies, the services accredited to conduct the procedures are generally staffed by experienced professionals who perform large numbers of operations annually, which is not always the case when procedures are performed sporadically or by less experienced professionals. In the case of endovascular surgery, specifically, there is also the factor of availability of the best and most suitable materials, which can make a difference to the results of angioplasty with stenting. Therefore, in the final analysis, for severe cases of symptomatic carotid stenosis, in which the risk of stroke is greater, it seems clear that there is no doubt that the choice of endarterectomy or angioplasty with stenting is consensus. In the case of severe asymptomatic carotid stenosis, while interventional procedures are also indicated in principle, there is room to consider several factors such as surgical risk, advanced age, female sex, and experience of the surgical team, among others, and to initially consider clinical treatment. 

 One subject that is often brought up when discussing the best form of treatment for severe asymptomatic carotid stenosis is the cognitive changes that are observed in patients with this disease. Since the incidence and risk factors of carotid atherosclerosis coincide with those of cerebral atherosclerosis, it is difficult to infer whether the cognitive deterioration observed in some patients who are treated clinically is caused by silent microembolizations and cerebral hypoperfusion or is the result of intracerebral lesions. On the other hand, it is also difficult to determine whether carotid revascularization can prevent cognitive deficits or even attenuate them. 4 While using transcranial Doppler to estimate encephalic reserves is effective, it has not proven to be a practical method for large scale evaluation. 

 One debate that is increasingly part of therapeutic decision-making in the context of improving imaging equipment and methods revolves around characterization of atheromatous plaques at the carotid bifurcation. Color Doppler mapping, angiotomography, and magnetic resonance imaging are all capable not only of providing morphological details, but also of indicating intraplaque hemorrhage or spontaneous embolization. However, there is no consensus on which of these diagnostic methods is best for characterization of carotid plaques and even less on their correlation with the degree of stenosis. While it is well-established that unstable (soft) plaques involve a greater risk of complications than stable (hard) ones, there is no consensus that this characteristic has greater predictive value for progression of carotid disease than the degree of stenosis. 5

 There are some clinical situations in which carotid stenosis may be associated with other serious cardiovascular diseases that also require surgical treatment, such as chronic coronary disease and abdominal aortic aneurysms. In such situations, it is important to define which procedure should be performed before the other. In general, the symptomatic lesion of greatest severity takes priority for surgery. In the case of open surgery for myocardial revascularization, intraoperative or postoperative stroke is rare, but when it does occur, mortality is significant. However, in this type of operation there is little evidence of a direct relationship between intraoperative or postoperative stroke and extracranial carotid stenosis, especially not in asymptomatic cases. Other factors, such as manipulation of the ascending aorta, cardiopulmonary bypass, or even prior injuries to intracerebral vessels appear to play the leading role in this situation. Indeed, recent guidelines from the European Society for Vascular Surgery on the subject do not recommend treating asymptomatic carotid stenosis either prior to or simultaneously with heart surgery, considering that only severe symptomatic carotid stenosis should be treated. As with many other aspects of carotid disease, this conduct cannot be considered consensus in the literature. With relation to abdominal aortic aneurysm repair, whether by open or endovascular surgery, there are no studies with sufficiently large patient samples to define whether coexisting carotid stenosis should be treated before or after aortic repair. There are reports that suggest that carotid stenosis that does not exceed 80% and is asymptomatic can be left until after aortic repair; but for stenosis exceeding 80%, there is no consensus. Once more, severe symptomatic carotid stenosis should be treated prior to aortic repair because of the increased risk of stroke. 6

 Cases of severe carotid stenosis in which there is contralateral carotid occlusion are also debatable in terms of therapeutic management. These patients are considered at greater risk of complications related to atherosclerotic disease, primarily cardiological, and if the non-occluded side is treated clinically, because of surgical risk, and the disease progresses, they may suffer severe neurological complications. If the decision to intervene is taken, it had been considered that since angioplasty with stenting does not occlude the artery during the procedure, it would involve a smaller risk of ischemic complications. However, studies comparing angioplasty with stenting with endarterectomy in this situation have not confirmed this reasoning and the majority of studies have found similar results for both techniques for asymptomatic stenosis, although endarterectomy has superior results for symptomatic stenosis. 7

 In the not-so-distant past, some authors recommended endarterectomy after diagnostic assessment exclusively with Doppler mapping, foregoing preoperative assessment of the intracranial circulation. However, as angiotomography and magnetic resonance imaging have become widespread, studies of the intracerebral arteries, and of the aortic arch and common carotid, have become part of routine preoperative assessment at the majority of specialist services, although the need for this assessment has not been demonstrated beyond doubt. In the case of indications for angioplasty with stenting, this assessment is part of the protocol for the procedure, with arteriography of the cerebral vessels both before dilation of the lesion at the carotid bifurcation and afterwards, as a control examination. 

 Another discussion that has been taking shape over recent years is related to the best time to intervene in patients with symptomatic carotid bifurcation lesions. The possibilities range from intervention immediately after diagnosis is confirmed, through 48 hours after a neurological event, from 48 hours to 1 week after, and even up to 2 weeks later. The justification for early intervention after a diagnosis of stroke is based on the idea of minimizing the effects of ischemia on the cerebral territory involved and avoiding a repeat stroke. However, revascularization during the acute or subacute phase of cerebral ischemia could possibly transform the ischemic effects on a given cerebral area into hemorrhagic effects, worsening the patient’s clinical status. A detailed assessment of neurological status and the clinical repercussions could provide a basis for choosing the best time for intervention, since there is still no consensus in the literature on each of the options. 8

 As we have seen, the controversies relating to management of patients with symptomatic or asymptomatic lesions of the bifurcation carotid are far from being resolved, despite almost 30 different sets of medical society guidelines aimed at organizing and standardizing conduct. Although there has been a perceptible movement over the last decade in the direction of reducing indications for intervention as a consequence of growing evidence of the efficacy of clinical treatment for asymptomatic stenosis, the legal and medical implications of not indicating carotid revascularization for a patient who fits the criteria for intervention according to current studies in the event that this patient has a stroke cannot be ignored. Ongoing, controlled, multicenter studies and future studies that are yet to be proposed may help to elucidate many of the controversial issues that remain. 
