# Supplementary material for: Carotid atherosclerotic disease
Source: J Vasc Bras. 2018 Jul-Sep;17(3):179–83. doi: 10.1590/1677-5449.011703 (PMC6326131; doi:10.1590/1677-5449.011703)
Supplement: Translation

# Translation: Doença aterosclerótica carotídea

**Authors:** Cid José Sitrângulo, Erasmo Simão da Silva

**Affiliations:** 1 Universidade de São Paulo – USP, Faculdade de Medicina, Disciplina de Cirurgia Vascular e Endovascular, São Paulo, SP, Brasil.

 O tratamento da doença aterosclerótica carotídea tem sido alvo de constantes debates e controvérsias ao longo do tempo, na medida em que novos conhecimentos e avanços tecnológicos vão sendo incorporados aos já previamente estabelecidos. Mesmo com um grande número de artigos científicos de boa qualidade publicados na literatura (trabalhos multicêntricos randomizados, séries de casos, revisões de literatura e metanálises, dados de grandes registros baseados na prática clínica real e opinião de *experts* sobre o tema), existe ainda margem para opiniões controversas sobre alguns aspectos da terapêutica clínica ou cirúrgica. Isso porque, do ponto de vista clínico, muitas considerações têm de ser feitas no momento da decisão de cada caso particular, em face da complexidade e variabilidade da doença em si e das possibilidades terapêuticas que se apresentam. Dessa forma, a estenose aterosclerótica da bifurcação carotídea é uma afecção cujas recomendações da medicina baseada em evidências por vezes esbarram na sensibilidade e experiência do terapeuta. 

 Inicialmente, seria interessante destacar que a estenose da bifurcação carotídea, foco principal deste artigo, é responsável pela ocorrência de aproximadamente 20% dos casos de isquemia cerebral. No entanto, existem relatos apontando que sua prevalência pode estar entre 6 e 40%, o que pode ser justificado pela existência de fatores concomitantes (doenças associadas) que contribuem para um expressivo número de eventos neurológicos de origem indeterminada. Além da estenose carotídea, o indivíduo pode ter doença cardíaca (fibrilação atrial, doença valvar, área inativa cardíaca pós-infarto agudo do miocárdio – IAM), doença do arco aórtico, doença das carótidas comuns, doença aterosclerótica intracerebral, entre outras. A prevalência da estenose carotídea aumenta com a idade e, aos 65 anos, 5% das pessoas têm estenose carotídea superior a 50%, sendo que os homens têm o dobro da incidência das mulheres. Com relação ao risco de portadores de estenose carotídea sofrerem acidente vascular cerebral (AVC), se a estenose for inferior a 50%, a chance é de menos de 1% ao ano, enquanto nas estenoses superiores a 50%, a chance é de 1 a 5% ao ano 1 . 

 É de relevante importância prognóstica a relação que existe entre estenose carotídea, doença aterosclerótica coronariana e doença arterial obstrutiva periférica dos membros inferiores. A estenose carotídea é considerada um importante marcador de risco cardiovascular e óbito de causa cardíaca, a tal ponto que os portadores de estenose carotídea progressiva têm maior risco iminente de desenvolver IAM do que futuros eventos cerebrais isquêmicos. Rothwell, em estudo prospectivo de pacientes que tiveram evento cerebral isquêmico, quantificou em 10 a 25% o risco de desenvolver IAM em 5 anos e de até 15% o risco de morte cardiovascular (exceto AVC) em 5 anos 2 . Por outro lado, com relação à severidade da estenose carotídea, embora não haja consenso, acredita-se que quanto maior o grau de estenose maior a possibilidade de evento neurológico. Da mesma forma, não há consenso se quanto pior o grau de estenose carotídea maior a possibilidade de manifestação de coronariopatia grave ou moderada. 

 As opções terapêuticas para a estenose carotídea aterosclerótica passam pelo tratamento clínico (*best medical therapy*), que implica um combate vigoroso dos principais fatores de risco para a aterosclerose (tabagismo, hipertensão arterial, diabetes e dislipidemia), associado à atividade física sistemática, dieta saudável e prescrição medicamentosa de antiagregantes plaquetários e estatinas em altas doses. Nos casos de lesões carotídeas mais acentuadas, pode se impor a realização de endarterectomia ou angioplastia e stent. No caso dessas modalidades intervencionistas de tratamento, vários fatores devem ser considerados, tais como idade, sexo, principais comorbidades, especialmente cardíacas, renais e pulmonares, além das condições anatômicas e patológicas do arco aórtico. Particularmente com relação à idade e ao sexo, algumas controvérsias existem sobre a melhor opção terapêutica. Nos pacientes idosos, geralmente, a tortuosidade arterial é maior e o processo aterosclerótico é mais intenso, provocando maior calcificação do arco aórtico e da própria carótida, aumentando, portanto, o risco de acidentes embólicos durante a angioplastia. No sexo feminino, em que a evolução natural da doença aterosclerótica pode ser mais agressiva e habitualmente o calibre das artérias é menor, existe a possibilidade aumentada de reestenose pós-operatória nos casos de endarterectomia, que constitui uma das recomendações de utilização de remendo arterial. 

 Os estudos controlados randomizados multicêntricos das décadas de 1980 e 1990 sobre estenoses carotídeas sintomáticas e assintomáticas, tanto nos Estados Unidos (NASCET – sintomáticas e ACAS – assintomáticas) quanto na Europa (ESCT – sintomáticas e ACST – assintomáticas), conduziram de alguma maneira à normatização da conduta frente às estenoses severas da artéria carótida interna. Esses estudos foram, de certa forma, concordantes em que tanto nos casos de estenoses sintomáticas quanto assintomáticas a endarterectomia era superior ao tratamento clínico na prevenção de eventos cerebrais isquêmicos futuros num período de 5 anos. 

 Com o desenvolvimento da cirurgia endovascular no território carotídeo, uma nova questão se tornou preponderante na prática clínica: a utilização da angioplastia e stent, procedimento sabidamente menos invasivo que a endarterectomia, produziria resultados semelhantes ou pelo menos não inferiores aos da endarterectomia? Com o objetivo de elucidar essa dúvida e fornecer novos subsídios para consolidar a indicação de cada uma dessas técnicas, novos estudos multicêntricos foram realizados nas décadas de 2000 e 2010 nos Estados Unidos (CREST foi o principal) e na Europa (ICSS, entre outros). Diferentemente dos estudos das décadas de 1980 e 90, desta vez os resultados dos principais estudos foram de alguma forma conflitantes: enquanto as conclusões do CREST mostraram resultados semelhantes tanto da endarterectomia quanto da angioplastia e stent na prevenção de eventos cerebrais isquêmicos futuros, uma boa parte dos estudos europeus concluiu pela inferioridade da angioplastia e stent em relação à endarterectomia, especialmente no caso das estenoses carotídeas sintomáticas. É interessante destacar que a Associação Americana de Cardiologia (AHA), inspirada fortemente nos resultados do CREST, divulgou suas diretrizes para o tratamento das estenoses carotídeas severas recomendando, de forma igualitária, tanto a endarterectomia quanto a angioplastia e stent. Apesar da contestação de autores europeus, a recente atualização das diretrizes da AHA manteve as mesmas recomendações iniciais. 

 Mais recentemente, vem sendo desenvolvido um novo estudo multicêntrico capitaneado pela Universidade de Oxford, no Reino Unido, e com a participação de vários centros universitários europeus, além do Serviço de Cirurgia Vascular e Endovascular do Hospital das Clínicas da Faculdade de Medicina da Universidade de São Paulo. O estudo ACST-2, que já conta com mais de 3.000 casos randomizados, compara os resultados da endarterectomia e da angioplastia e stent nos casos de estenose carotídea assintomática na prevenção de eventos isquêmicos cerebrais futuros. 

 Paralelamente a toda essa discussão sobre a melhor forma de tratamento intervencionista da estenose carotídea severa, vem ocorrendo nos últimos anos, na “vida real”, uma diminuição das indicações tanto de endarterectomia como de angioplastia e stent na condução desses casos. A explicação mais plausível para esse fato é o reconhecimento, especialmente por parte dos clínicos, cardiologistas e neurologistas, de que o tratamento clínico ( *best medical therapy*), atualmente proposto de forma mais agressiva e completa, quando conta com a adesão dos pacientes, pode diminuir a ocorrência de AVC, especialmente nas estenoses carotídeas assintomáticas. Muitos autores respeitados na literatura internacional têm se manifestado nesse sentido. Para reforçar essa convicção, podemos nos reportar aos trabalhos que documentaram através de exames de imagem (mapeamento Doppler, ressonância magnética e angiotomografia) a estabilidade da placa de ateroma ou até mesmo sua regressão com o uso de altas doses de estatina e controle rigoroso dos fatores de risco para a aterosclerose 3 . Devemos levar em consideração o fato de que, nos grandes estudos iniciais sobre estenose carotídea, a utilização das estatinas era irregular e heterogênea. Atualmente, por outro lado, outras drogas complementares também podem ser associadas, quando necessárias, para o controle do colesterol e triglicérides (ezetimibe, fibratos, ácido nicotínico), e esse pode ser um fator crucial para produzir resultados mais satisfatórios do tratamento clínico. Entre os antiagregantes plaquetários, a aspirina é a mais utilizada, embora exista a opção do clopidogrel ou da ticlopidina. O controle da pressão arterial conta com a utilização de drogas hipotensoras com componentes de proteção endotelial, como inibidores das enzimas de conversão da angiotensina ou inibidores da angiotensina. O controle precoce de diabetes e a cessação do tabagismo são fundamentais, assim como a modificação dos hábitos de vida com promoção da atividade física regular e permanente. Com esse conjunto de medidas terapêuticas integradas, a progressão da doença aterosclerótica pode ser contida, reduzindo seus efeitos sobre o território carotídeo e cardíaco e justificando a constatação de que, atualmente, o tratamento clínico é mais eficiente que no passado. Adicionalmente, os benefícios da endarterectomia sobre o tratamento clínico, demonstrados nos estudos iniciais das lesões carotídeas assintomáticas, sempre foram limítrofes e baseados em taxas mais altas de AVC com terapias clínicas menos incisivas. Dessa forma, principalmente para os pacientes de alto risco clínico ou cardiológico, o tratamento clínico da estenose carotídea severa assintomática vem se tornando mais atraente. 

 Por outro lado, o tratamento clínico otimizado é desrespeitado com frequência, visto que a aderência é variável por parte de clínicos e pacientes. Além disso, a mudança no estilo de vida é difícil de ser atingida (parar de fumar, comer bem, emagrecer, realizar exercícios). O número de medicações para tratamento dos fatores de risco é grande e necessita de titulação constante da dose, o que requer várias visitas ao clínico. A estrutura de consulta ideal com profissionais de nutrição, psicologia (parar de fumar) e enfermagem para pré e pós-consulta é preconizada e seguida nos estudos controlados randomizados, mas nem sempre na “vida real”, especialmente nos países pobres. 

 Um outro debate interessante diz respeito à reprodutibilidade dos resultados dos estudos controlados em relação aos da “vida real”. É notório que os resultados dos procedimentos invasivos realizados fora dos estudos randomizados e controlados são inferiores. É intuitivo também que diferentemente dos estudos controlados, nos quais os pacientes são alocados para tratamento segundo padrões rígidos e uniformes de seleção, na prática clínica diária, muitos fatores que limitariam a inclusão de pacientes em estudos estão presentes e podem ser responsáveis por mais complicações operatórias e pós-operatórias, levando a resultados inferiores aos dos estudos controlados. Além disso, nos estudos controlados, os serviços credenciados para realizar os procedimentos geralmente contam com profissionais experimentados e com grande número anual de operações, o que nem sempre é verdadeiro quando os procedimentos são feitos de forma esporádica ou por profissionais menos experientes. No caso da cirurgia endovascular, em particular, existe ainda o detalhe da disponibilidade dos melhores e mais adequados materiais que podem diferenciar também os resultados da angioplastia e stent. Portanto, em última análise, nos casos das estenoses carotídeas severas sintomáticas, em que o risco de AVC é maior, parece não haver dúvida de que a indicação de endarterectomia ou angioplastia e stent é consensual. Já no caso das estenoses carotídeas severas assintomáticas, embora em princípio o procedimento intervencionista também esteja indicado, há margem para ponderar vários aspectos como risco cirúrgico, idade avançada, sexo feminino, experiência da equipe cirúrgica, entre outros, no sentido de considerar inicialmente o tratamento clínico. 

 Um aspecto que frequentemente vem à tona quando se discute sobre a melhor forma de tratamento das estenoses carotídeas severas assintomáticas diz respeito às alterações cognitivas dos pacientes acompanhados com essa doença. Como a incidência e os fatores de risco da aterosclerose carotídea coincidem com a da aterosclerose cerebral, é difícil inferir se a piora cognitiva observada em alguns pacientes tratados clinicamente é decorrente de microembolizações silenciosas e hipoperfusão cerebral ou resultado das lesões intracerebrais. Por outro lado, também é difícil avaliar se a revascularização carotídea pode prevenir déficits cognitivos ou até mesmo atenuá-los 4 . A utilização do Doppler transcraniano para estimar a reserva encefálica, embora eficaz, não tem se mostrado um método prático de avaliação em larga escala. 

 Uma discussão que cada vez mais ocupa espaço na decisão terapêutica em face do progresso dos equipamentos e métodos de imagem é a caracterização da placa de ateroma da bifurcação carotídea. Tanto o mapeamento Doppler colorido quanto a angiotomografia e também a ressonância nuclear magnética podem fornecer não só detalhes morfológicos como até mesmo indícios de hemorragia intraplaca ou embolização espontânea. No entanto, não há consenso sobre qual o melhor desses métodos de diagnóstico para a caracterização da placa carotídea, muito menos sua correlação com o grau de estenose. Embora esteja bem estabelecido que as placas instáveis (moles) apresentem maior risco de complicação que as estáveis (duras), não há consenso de que essa característica tenha valor preditivo maior que o grau de estenose na evolução da doença carotídea 5 . 

 Existem algumas situações clínicas em que a estenose carotídea pode estar associada a outras doenças cardiovasculares graves e que também requerem tratamento cirúrgico, como a coronariopatia crônica e o aneurisma da aorta abdominal. Nessas eventualidades, é preciso definir qual procedimento deve preceder o outro. De maneira geral, a lesão sintomática de maior gravidade tem preferência na indicação cirúrgica. No caso da cirurgia aberta de revascularização do miocárdio, a ocorrência de AVC intra ou pós-operatório é rara, mas, quando acontece, cursa com importante mortalidade. Entretanto, nesse tipo de operação, não há evidências da relação direta do AVC intra ou pós-operatório com a estenose carotídea extracraniana, principalmente nos casos assintomáticos. Outros fatores como manipulação da aorta ascendente, do *bypass* cardiopulmonar, ou mesmo lesões prévias dos vasos intracerebrais parecem ter papel preponderante nessa situação. Tanto é que nas recentes diretrizes da Sociedade Europeia de Cirurgia Vascular sobre esse assunto não há recomendação de tratar as estenoses carotídeas assintomáticas previamente ou simultaneamente à cirurgia cardíaca, considerando passíveis de tratamento prévio apenas as estenoses carotídeas severas sintomáticas. Como em muitos outros aspectos da doença carotídea, essa conduta não pode ser considerada consensual na literatura. Já na correção do aneurisma da aorta abdominal, por via aberta ou endovascular, não existem trabalhos com casuística suficiente para definir se a estenose carotídea associada deva ser tratada antes ou após a correção aórtica. Há trabalhos que sugerem que estenoses carotídeas inferiores a 80%, assintomáticas, podem aguardar a correção aórtica; porém, para as estenoses superiores a 80%, não há consenso. Também aqui as estenoses carotídeas severas sintomáticas devem ser tratadas previamente à correção aórtica devido ao risco aumentado de AVC 6 . 

 Os casos de estenose carotídea severa associada à oclusão carotídea contralateral também são passíveis de discussão quanto à condução terapêutica. Considerados portadores de maior risco de complicações relacionadas à doença aterosclerótica, principalmente cardiológicas, esses pacientes, se tratados de forma clínica no lado não ocluído, devido ao risco cirúrgico, podem, em caso de progressão da doença, evoluir com graves complicações neurológicas. Se a opção for pela intervenção, tem sido considerada a ideia de que a angioplastia e stent, por não ocluir a artéria durante o procedimento, teria menor risco de complicações isquêmicas. No entanto, trabalhos comparando a angioplastia e stent com a endarterectomia nessa situação não têm confirmado esse raciocínio, mostrando, na maioria dos estudos, resultados semelhantes para as duas técnicas nas estenoses assintomáticas, porém superiores para a endarterectomia nas estenoses sintomáticas 7 . 

 Num passado não muito distante, alguns autores preconizavam a realização da endarterectomia após avaliação diagnóstica exclusivamente com o mapeamento Doppler, dispensando, portanto, a avaliação pré-operatória da circulação intracraniana. Com o emprego mais disseminado da angiotomografia e da ressonância magnética, o estudo das artérias intracerebrais, assim como do arco aórtico e da carótida comum, passou a fazer parte da rotina de avaliação pré-operatória na maioria dos serviços especializados, embora a comprovação dessa necessidade de avaliação prévia não tenha sido demonstrada de forma cabal. No caso da indicação da angioplastia e stent, essa avaliação faz parte do protocolo do procedimento, tanto através da arteriografia dos vasos cerebrais antes da dilatação da lesão da bifurcação carotídea como após a sua realização, como controle. 

 Uma discussão que vem ganhando corpo nos últimos anos diz respeito ao momento mais adequado para a intervenção no paciente com lesão da bifurcação carotídea sintomática. As possibilidades vão desde intervir imediatamente após o diagnóstico confirmado, depois de 48 horas do evento neurológico, entre 48 horas e uma semana, ou mesmo após duas semanas. A justificativa para a intervenção precoce após o diagnóstico de AVC encontra respaldo na ideia de minimizar os efeitos da isquemia no território cerebral acometido e evitar novo AVC. Entretanto, a revascularização na fase aguda ou subaguda da isquemia cerebral pode eventualmente transformar os efeitos isquêmicos de determinada área cerebral em hemorrágicos, com agravamento do estado clínico do paciente. A avaliação minuciosa do quadro neurológico e suas repercussões clínicas pode orientar quanto ao melhor momento para intervir, uma vez que não há ainda consenso na literatura sobre cada uma dessas possibilidades 8 . 

 Como vemos, as controvérsias sobre como conduzir os pacientes com lesões sintomáticas ou assintomáticas da bifurcação carotídea estão longe de serem dirimidas, a despeito de quase 30 diretrizes de sociedades médicas tentarem organizar e padronizar as condutas. Embora haja um movimento percebido na última década de diminuição de indicação de intervenção em consequência do fortalecimento das evidências da eficácia do tratamento clínico nas estenoses assintomáticas, não se pode desprezar a preocupação médico-legal de não se indicar a revascularização carotídea num paciente que se enquadre nos critérios de indicação de intervenção segundo os estudos vigentes, e esse paciente vir a sofrer um AVC. Os estudos controlados multicêntricos vigentes sobre o assunto e novos que eventualmente sejam propostos poderão colaborar para o esclarecimento de muitos pontos polêmicos ainda existentes.
